# Ultra-weak photon emission: from oxidative metabolism to DNA-based communication: a review of biochemical, biophysical and quantum biological perspectives

**DOI:** 10.3389/fendo.2026.1861061

**Published:** 2026-08-20

**Authors:** Jozsef Bodis, Jozsef Berke, Bernadett Nagy, Istvan Gulyas, Péter Hersics, Akos Varnagy, Kalman Kovács

**Affiliations:** 1 National Laboratory on Human Reproduction, University of Pécs, Pecs, Hungary; 2 Department of Obstetrics and Gynecology, School of Medicine, University of Pécs, Pecs, Hungary; 3 UltraSmart LTD, Érd, Hungary

**Keywords:** biophoton, DNA communication, embryo assessment, quantum biology, ultra-weak photon emission

## Abstract

**Introduction:**

Ultra-weak photon emission (UPE) from living systems has been reported and linked to oxidative reactions. Whether photons mediate communication—particularly at the level of DNA—remains unresolved. Rather than interpreting UPE solely as a metabolic byproduct, increasing evidence suggests that it represents a structured, state-dependent optical correlate of cellular organization. To review biochemical and quantum-biological bases of UPE, summarize measurement approaches, and evaluate whether DNA-related emission could support signalling, and critically evaluate whether DNA-related emission may have functional relevance for biological signalling.

**Methods:**

We synthesised literature on sources (reactive oxygen species, lipid peroxidation, protein/DNA oxidation) and detectors (photomultiplier tubes, cooled CCD cameras, Complementary Metal-Oxide Semiconductor CMOS). We measured UPE from mouse embryos in a dark incubator using an ORCA-Quest CMOS system.

**Results:**

UPE is modulated by cellular state; mitochondria, membranes and peroxisomes are key contributors. Models posit DNA as a storage/emitter and potential resonator. Several theoretical models suggest that DNA may contribute to photon emission dynamics and resonance-like behaviour under specific conditions, although functional interpretations remain under investigation.

**Discussion:**

Ultra-weak cellular photon emission—especially the proposed DNA-linked mechanisms—remains a challenging yet promising field. Evidence does not convincingly show DNA acts as a biophoton communication system. Current evidence does not conclusively demonstrate that DNA functions as a dedicated biophoton communication system but the hypotheses suggest new ways to view biological information processing and cellular function. Importantly, structured emission patterns and their dependence on cellular state suggest that UPE may represent an optical correlate of higher-order biological organization. Importantly, integrating UPE measurements into embryology may provide novel insights into reproductive biology and assisted reproduction, offering a potential non-invasive biomarker for embryo selection and developmental potential.

## 1 Introduction

Photon emission by living cells – also known as biophoton emission – has received increasing attention in the fields of biophysics, biochemistry, and quantum biology in recent decades (1–3). This phenomenon refers to extremely low-intensity (ultra-weak) light emitted by living organisms under natural conditions without external excitation, which falls within the visible and near-ultraviolet range (approx. 200–800 nm) (4–9) Although it is not visible to the human eye, it can be detected and characterized with sufficiently sensitive detectors. Several research groups (10–14) have begun to conduct research on the ultra-weak photon emission of biological systems by the use of ultra-low noise, highly sensitive photon counting systems. Beyond its biophysical significance, ultra-weak photon emission (UPE) may have important implications for endocrine regulation, particularly in reproductive processes where redox balance, mitochondrial activity, and embryo viability are tightly linked to hormonal signalling. Recent studies increasingly suggest that UPE is not merely a passive byproduct of metabolism but may represent a measurable, state-dependent optical correlate of cellular activity and organization.

**Figure 1:**
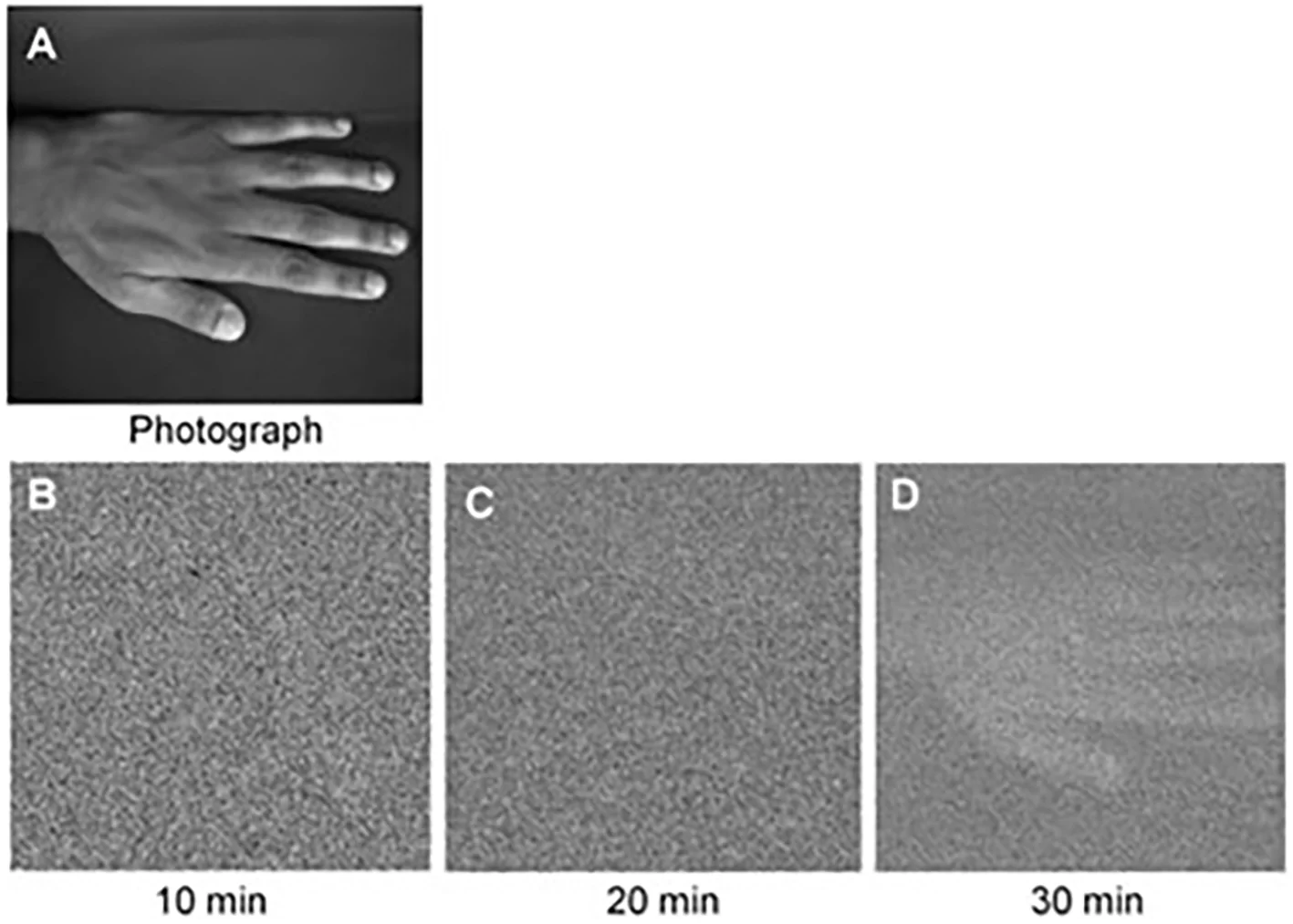
Two-dimensional imaging of spontaneous ultra-weak photon emission from the human hand. The photograph **(A)** taken under weak light illumination and the corresponding two-dimensional images of spontaneous ultra-weak photon emission with variable accumulation time of 10 min **(B)**, 20 min **(C)** and 30 min **(D)**. The dorsal side of the human hand was positioned at 37 cm from CCD camera window. Figure reproduced without modification from: Prasad and Pospíšil (103). This work is licensed under a Creative Commons. Attribution-NonCommercial-NoDerivs 3.0 Unported License.

The first systematic observations of photon emission are attributed to Alexander Gurwitsch, who discovered in the 1920s that cells emit light at certain stages of development, which can induce the division of other cells. He called this phenomenon “mitogenic radiation (15).” The concept was controversial for a long time, the concept remained controversial for several decades, but in the 1970s, Fritz-Albert Popp and his colleagues used new measurement techniques to confirm that living cells do indeed produce ultra-weak photon emission, and they hypothesized that this emission could be interpreted as a coherent quantum process, and proposed that this emission may exhibit coherent-like properties under certain conditions (11). Biophoton emission has now been observed in many cell and tissue types, including plants, animals, bacteria, human cell lines and body (7, 9, 16–22) (**Figure 1**).

Du, J. et al. in their review collected experimental findings showing that UPE intensity changes in diabetes, cancer, neurodegeneration, and cardiovascular diseases (23). Several studies reported that oxidative stress biomarkers strongly correlate with photon emission levels. The paper described successful applications of CCD cameras and photomultiplier systems for imaging metabolic activity in tissues. The authors highlighted that photon emission patterns can vary according to circadian rhythms, aging, and therapeutic interventions. They concluded that UPE analysis could become a practical clinical tool for monitoring disease progression and treatment response. These findings support the interpretation of UPE as a functional indicator of cellular physiological state rather than purely stochastic oxidative emission.

Nevoit, G., et al. reviewed experimental evidence suggesting that neurons and other cells emit ultra-weak photons during metabolic and electrical activity (24). The authors discussed studies reporting correlations between photon emission and brain function, oxidative metabolism, and mitochondrial activity. They analysed theoretical models proposing that photons could contribute to intracellular or intercellular signalling. They discussed theoretical models proposing that photon emission may reflect coordinated intracellular or intercellular dynamics. However, they emphasized that direct proof of functional biophotonic communication in the brain is still lacking. The article concluded that more sensitive detection methods and reproducible experiments are necessary to test the signalling hypothesis.

The emission is typically associated with oxidative biochemical reactions (e. g., the formation of reactive oxygen species) and is sensitive to various physiological and pathological processes.

A growing body of research suggests a potential biological role for photon emission, including intra- and intercellular communication, regulation of cellular processes, and fine-tuning of environmental responses. In addition, quantum biological interpretations have emerged suggesting that photon fields, coherent radiation, and quantum fluctuations may also operate in cells, promoting self-organization and communication.

The aim of this review is to:

1. Systematize the biochemical and biophysical basis of biophoton emission.
2. Present the experimental methods currently in use and their limitations.
3. Evaluate quantum biological theories and their empirical basis.
4. Review the possibilities of cell-level and intercellular light-based communication.
5. Present scientific evidence for photon emission as a means of DNA communication.
6. Identify currently open questions and suggest directions for further research.

## 2 Biological mechanisms of photon emission

### 2.1 Biochemical background

Biophotons emitted by living cells are generally considered to originate primarily from metabolic processes. with particular emphasis on oxidative and peroxidative reactions. According to the most widely accepted explanation, biophoton emission is the result of the relaxation of excited states (e. g., triplet oxygen, singlet oxygen, excimer complexes) generated during redox reactions in cells. Early interpretations of biophoton research predominantly considered photon emission as a metabolic byproduct. However, advances in biophotonics and related interdisciplinary fields have led to a more nuanced understanding of this phenomenon. Recent perspectives suggest that photon emission may not be solely stochastic but can reflect structured, state-dependent biological processes.

The main sources of endogenous photon emission arise from several oxidative processes within biological systems. A major contributor is the formation of reactive oxygen species (ROS), such as superoxide anion (O_2_^–^), hydrogen peroxide (H_2_O_2_), and singlet oxygen (¹O_2_), which are highly reactive and short-lived molecules (25–28). Through their interactions with cellular components, these ROS generate electronically excited species that release photons as they return to their ground state.

Recent theoretical developments have shifted the interpretation of UPE from a passive metabolic byproduct toward the broader concept of biophotonic signalling, in which structured photon emission reflects dynamic cellular organization rather than solely oxidative chemistry (24).

Lipid peroxidation also represents a significant source of photon emission. During the oxidation of unsaturated fatty acids, various by-products are formed—most notably malondialdehyde and other aldehyde derivatives—which participate in chemical reactions that produce excited intermediates capable of emitting light (29).

**Figure 2:**
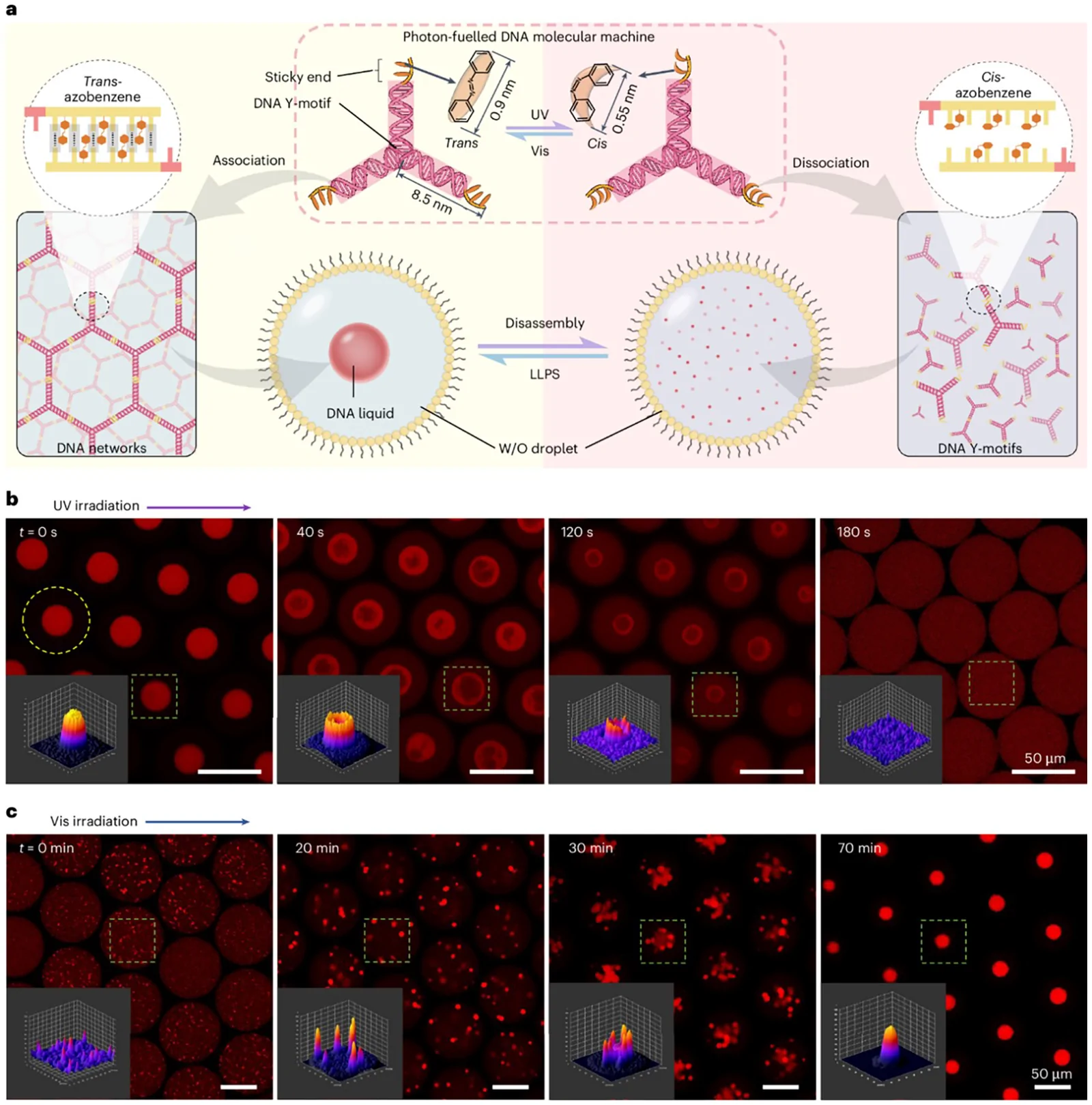
Design of Y-shaped DNA nanomachines with azobenzenes as nano-energy converters and photoinduced reversible assembly of DNA droplets. In Vis light, DNA nanomachines assemble into DNA droplets, whereas in UV light, DNA droplets dissociate. **(b)**, CLSM images showing the disassembly processes of DNA droplets (Cy5 stain) in W/O droplets on UV irradiation. **(c)**, CLSM images showing the reassembly processes of DNA droplets in Vis irradiation. Insets showing the fluorescence intensity profiles in the dashed boxes. Scale bars, 50 μm. Figure reproduced without modification from: **Figure 1** of B Zhao et al., 2025 “DNA photofluids show life-like motion.” Nature Materials 24(6):935-944, April 2025.

In addition, the oxidation of proteins and DNA contributes to photon generation (**Figure 2**). These oxidative processes yield free radicals and electronically excited molecular fragments that emit photons during their relaxation back to the ground state (30). Importantly, while these processes are fundamentally stochastic at the molecular level, emerging experimental evidence suggests that the resulting photon emission may exhibit non-random temporal dynamics at the cellular level.

During these processes, chemical energy creates electron-excited states, which release photons in the visible or near-UV range during de-excitation. The intensity of the emitted photons is extremely low – typically 1–100 photons/s/cm² – and can therefore be measured only under specially shielded conditions and with highly sensitive detectors. The emission is not uniform and may exhibit stochastic fluctuations as well as quasi-periodic or structured temporal patterns.

Numerous studies have observed that the intensity and spectrum of the emission are sensitive to changes at the cellular level: for example, they increase in the case of oxidative stress, apoptosis, cell proliferation, or inflammatory response (31–33).

Mould, R. R., and his coworkers in their review summarized evidence that ultra-weak photon emission increases during oxidative stress, inflammation, and metabolic activation (34). Multiple studies cited in the publication showed elevated photon emission in cancer cells, stressed plants, and damaged tissues. They explained that reactive oxygen species and lipid peroxidation are major biochemical sources of these photons and concluded that UPE has potential as a non-invasive indicator of physiological and pathological processes.

Nowak, M et al. exposed crowén ethers to fenton reagents and detected strong ultra-weak photon emission generated during oxidative radical reactions (35). They observed that photon intensity depended on the molecular structure of the crown ethers and reaction conditions. Spectral analyses indicated that excited oxygen species contributed to photon generation. The experiments confirmed that hydroxyl radical-mediated oxidation can produce detectable biophotonic signals. The study provided a physicochemical model for understanding photon emission during oxidative stress reactions in biological systems. Together, these findings reinforce the view that biochemical reactions provide the primary source of photon emission, while the observed emission patterns may carry additional information about the underlying cellular state.

### 2.2 Photon emission at the cellular level

Photon emission within cells is not uniformly distributed; instead, it predominantly originates from specific intracellular structures. One of the main sites is the mitochondria, where large quantities of reactive oxygen species are generated during oxidative phosphorylation. Within the mitochondrial electron transport chain, several intermediates can form that are capable of emitting photons directly (34, 36, 37).

Another major source of photon emission is the cell membrane and various intracellular membrane systems. These structures are especially active in this regard because lipid peroxidation—a key process involved in ultra-weak photon generation—occurs extensively within membrane lipids (28, 38–47).

Popp proposed that biophotons may originate from DNA and be concentrated within the cell nucleus, suggesting that biological systems may store and release photon energy over time (11). While this hypothesis has influenced subsequent theoretical work, its functional interpretation remains a subject of ongoing investigation.

The emission pattern and intensity are closely related to the metabolic activity, cell cycle, and stress state of the cell. For example, it has been observed that photon emission increases significantly before cell division or during apoptosis (48). It is interesting to note that in some cases, the emission follows rhythmic patterns, suggesting the role of internal oscillators or cyclic cell regulatory systems.

Paolis, L.D. et al. analysed experimental biophoton signals and found long-range temporal correlations and non-random fluctuations in photon emission (49). Their statistical analyses suggested that photon production is not purely caused by random oxidative reactions but may reflect coordinated biological dynamics. Scaling behaviours have been observed similar to those seen in complex systems near criticality. They also discussed the possibility that biophoton emissions may reflect underlying cellular organization and adaptive processes. They concluded that current biochemical explanations alone cannot fully explain the observed coherence and temporal structures of the signals.

Prasad, A. and his coworkers showed that mechanical injury in plants rapidly increased ultra-weak photon emission intensity (50). Spectral measurements revealed enhanced emission in wavelength ranges associated with reactive oxygen species activity. The strongest photon signals appeared immediately after wounding and gradually decreased during recovery. The authors linked these emissions to lipid oxidation and stress-response pathways activated in damaged tissues. Their findings demonstrated that UPE can monitor real-time physiological responses to injury in living plants. These observations support the concept that photon emission is closely linked to physiological state changes and may function as a real-time indicator of cellular stress and adaptation.

Several open questions remain regarding cellular photon emission. Which specific molecular reactions are responsible for the highest photon emission, and is it possible to selectively inhibit or enhance those reactions? To what extent is photon emission dependent on cell type, and are there particular cell lines that emit light especially strongly or with regular patterns? How does the internal architecture of the cell—for example, the spatial distribution of mitochondria—shape the observed emission pattern? Finally, is it feasible to perform real-time, spectrally selective mapping of photon emission from cells both *in vitro* and *in vivo*?

## 3 Experimental approaches and challenges

Measuring biophoton emission is a technologically extremely challenging and complex task, as the emission intensity is several orders of magnitude lower than ambient light or classical fluorescent signals. Accordingly, research is based on high-sensitivity detection, ensuring a dark environment, and precise handling of samples. In particular, recent publications have highlighted both experimental and epistemological aspects of UPE research, emphasizing the need for standardized methodologies and reproducible experimental frameworks (51, 52).

The contemporary investigation of ultra-weak photon emission (UPE) is supported by a rapidly evolving methodological framework. Recent reviews have highlighted that UPE research has progressed from early observational studies toward highly standardized physical detection approaches employing photomultiplier tubes (PMTs), charge-coupled device (CCD) cameras, electron-multiplying CCD systems, spectroscopic techniques, and advanced digital signal-processing methods. These developments have substantially improved sensitivity, spectral characterization, and reproducibility of photon measurements in biological systems (51).

In parallel, broader analyses of the field have emphasized the historical and conceptual evolution of biophoton research, describing the transition from exploratory observations to a multidisciplinary scientific discipline integrating physics, chemistry, biology, and systems medicine. This methodological maturation has strengthened the scientific basis for investigating UPE as a potential indicator of metabolic activity, oxidative processes, and intercellular signalling phenomena (52).

The present review builds upon these methodological advances and focuses specifically on the biological significance of UPE, particularly its relationship to reactive oxygen species generation, mitochondrial activity, and endocrine–reproductive regulation”.

### 3.1 Measurement techniques

#### 3.1.1 Photon detection systems

Photon emission from biological samples is measured using several highly sensitive detection systems. The most widely applied instruments are photomultiplier tubes (PMTs), which are capable of detecting individual photons with exceptionally low background noise. Their main limitation is that they generally provide only integrated intensity measurements within a selected wavelength range and do not offer spatial resolution (53, 54).

Another important class of detectors is cooled CCD cameras, which allow imaging-based photon detection. This makes it possible to examine cell- or tissue-specific patterns of photon emission. Deep cooling, often to around –80 °C, substantially reduces dark noise; however, these cameras typically suffer from relatively low temporal resolution (55–58).

More recent developments include single-photon avalanche diodes (SPADs), which enable photon-by-photon, time-resolved measurements and can be incorporated into compact systems such as lab-on-a-chip platforms. Their major strength is excellent temporal resolution, though they exhibit wavelength-dependent sensitivity (59). Due to their high temporal resolution and discrete detection capabilities, SPAD sensors facilitate detailed analysis of photon emission dynamics. These properties make them particularly suitable for investigating temporal structure and potential non-random characteristics of UPE signals.

Finally, CMOS (Complementary Metal-Oxide Semiconductor) detectors represent another technological direction, offering an alternative architecture with potential for integration into miniaturized and high-speed imaging systems.

A key technological advantage as well as a weakness of sCMOS is that each pixel has its own read-out circuitry which introduces some variance in low-light level imaging and any electron-multiplication. Repeating this process for every pixel leads to a significant variance and increased background noise (60).

#### 3.1.2 Environmental and measurement conditions

Accurate detection of ultra-weak photon emission requires carefully controlled environmental and measurement conditions. Experiments must be performed in a fully isolated darkroom, where all external photons are excluded. Such facilities often employ multi-layer optical shielding, vibration-free flooring, and precise thermostatic regulation to ensure stable measurement conditions.

Proper calibration of the detection system is also essential. The exact dark count and spectral response of the detector must be known, typically determined through dark-frame–based procedures in which the intrinsic sensor noise and its temperature dependence are measured without any sample present. Importantly, these calibration steps should be performed independently of acquisition software, when possible, in order to minimize software-related bias.

Finally, strict control of temperature and pH is critical, as cellular metabolism—and therefore photon emission—is extremely sensitive to these environmental parameters. Even small fluctuations can significantly alter the measured signal.

### 3.2 Samples and experimental protocols

#### 3.2.1 Typical models for measurements

A variety of biological models are used to investigate ultra-weak photon emission. *In vitro* cell cultures are among the most common, as they offer a standardized and highly controlled environment; frequently used examples include HeLa cells, fibroblasts, and various plant cell lines. More specialized approaches employ isolated cell organelles, such as mitochondria or membrane fragments, which allow targeted biochemical analyses of specific photon-emitting structures. For studies aimed at understanding physiological relevance, researchers often turn to tissue samples or whole living organisms, including zebrafish embryos, mouse embryos, or Arabidopsis leaves, which provide insight into photon emission in complex biological systems.

Yang, M. et al. measured photon emission from patients with type 2 diabetes before and after traditional Chinese medicine treatment (61). They found significant reductions in photon emission intensity following therapy, especially in body regions associated with metabolic regulation. The decrease in UPE correlated with improvements in blood glucose levels and clinical symptoms. The study suggested that oxidative stress levels were reduced after treatment. The authors concluded that UPE measurements may provide a non-invasive method for evaluating therapeutic effectiveness in metabolic disorders.

Sample preparation plays a crucial role in ensuring the validity of measurements. Mechanical stress, exposure to oxidative conditions, or disruptions to cellular metabolism can artificially elevate photon emission, leading to misleading results. Therefore, careful handling and controlled preparation protocols are essential.

#### 3.2.2 Challenges and limitations

Despite technological advances, several challenges continue to limit the reliability and comparability of photon emission measurements. One major difficulty is the low signal-to-noise ratio: even under optimized conditions, distinguishing true biological photon emission from background noise remains problematic. A further limitation is the lack of standardized measurement procedures across research groups, which complicates direct comparison of experimental results. Environmental factors also introduce variability. Temperature, humidity, and electromagnetic noise can all influence detector performance and thereby affect measurement accuracy.

In addition, inherent biological variability poses a significant challenge, as cellular heterogeneity and dynamic metabolic states can reduce reproducibility and complicate interpretation. Together, these limitations emphasize the need for harmonized protocols, improved detector technologies, and integrative analytical approaches to fully exploit the potential of UPE measurements.

## 4 Quantum biological interpretations

However, subsequent theoretical approaches—particularly those of Fritz-Albert Popp and colleagues—suggested that this phenomenon might reflect more than passive oxidative processes, potentially involving structured emission and coherence-like properties. In contemporary interpretations, these ideas are increasingly framed within the concept of biophoton signalling, which does not necessarily imply direct communication but rather coordinated physical dynamics associated with biological systems.

### 4.1 The coherent biophoton theory

According to Popp and his colleagues (62, 63), biophoton emission should not be regarded as random background noise, but rather as a quasi-coherent electromagnetic field. In this view, the photon field may display low entropy characteristics, suggesting that temporal and spatial emission patterns may deviate from purely random distributions. The emission spectrum may even contain monochromatic components, particularly during specific physiological processes. Within this framework, cells behave as quantum oscillators capable of generating light pulses and potentially synchronizing their emission patterns with one another.

Popp interpreted these photons using analogies to coherent light sources such as lasers, where molecules present in cells can be collectively excited, and emission can be induced. This idea is consistent with the concepts of quantum optical coherence and super radiance.

Benfatto, M. et al. measured ultra-weak photon emission from biological samples using highly sensitive photomultipliers and detected intermittent bursts of photons rather than constant emission (64). Statistical analysis showed deviations from Poisson distributions, indicating that the photon signals were not random noise. They identified memory effects and long-time correlations in the emission patterns. Their data suggested the existence of collective intracellular processes influencing photon production. The authors proposed that these emission dynamics may reflect coordinated or self-organized biological activity.

### 4.2 Quantum coherence and quantum information in living systems

In recent years, the field of quantum biology has gained increasing attention, driven by several notable examples. In photosynthesis, light energy can be transferred in a quantum coherent manner within photosynthetic complexes, such as FMO complexes, as confirmed by femtosecond spectroscopy (65). Studies on bird navigation have revealed signs of geomagnetic sensing based on quantum spin entanglement in the retina of redstarts (66, 67). Additionally, in the context of olfaction, some theories propose that odour molecules activate receptors via quantum tunnelling mechanisms (68–70).

Building on these findings, it has been hypothesized that biophoton emission in living systems may involve quantum-consistent processes, although direct experimental evidence remains limited. In this framework, photons may represent physical carriers of energy in cellular systems rather than confirmed communication signals. Cells could behave as a quantum network, with electromagnetic fields synchronizing cellular activity across the system. Moreover, photon emission may follow nonlinear dynamics, potentially manifesting in complex or fractal-like temporal patterns (48).

### 4.3 Critical remarks and methodological issues

While quantum biological interpretations of biophoton emission are intriguing, several challenges remain. One major limitation is the coherence time: biological systems operate in a thermal, noisy environment, where the duration of quantum coherence is extremely short, typically on the order of picoseconds. Furthermore, there is a lack of direct empirical evidence, as few measurements have conclusively demonstrated coherent biophoton emission. Experimental data are highly sensitive to background noise, and statistical processing must be conducted with great caution to avoid artifacts. Importantly, many observed patterns in photon emission can also be explained by classical nonlinear processes, such as oscillations or stochastic resonance, highlighting the need to consider alternative, non-quantum explanations. This underscores the importance of cautious interpretation and the avoidance of overextending quantum analogies beyond available experimental evidence.

Nevertheless, these interpretations provide a conceptual framework that may guide future interdisciplinary research. To move forward, multidisciplinary experimental protocols combining quantum optical, biophysical, and biochemical methods are needed.

## 5 Light-based communication at the cellular and intercellular levels

Biophoton emission can be interpreted not only as a by-product of the metabolic state of cells, but also, based on numerous experimental observations, as a potential indicator of biologically relevant information. According to this concept, photon emission patterns may be associated with coordinated intracellular or intercellular processes. This phenomenon raises the possibility of a new type of non-molecular signal transmission channel. However, the functional relevance of such photon-based mechanisms remains under active investigation.

### 5.1 Intracellular light signals

Intracellular photon emission is primarily associated with specific cellular structures. Mitochondria are a major source, as reactive oxygen species (ROS) generated during cellular respiration and their subsequent chemical reactions can produce photons. These so-called “internal light points” may reflect temporal and spatial patterns associated with intracellular activity (71). Another proposed contributor is the microtubule network; some theories suggest that the hollow internal structure of microtubules may enable photon conduction, effectively acting as “biological optical fibers” (72). This concept has been discussed in connection with broader theoretical models of intracellular signal propagation (73). Additionally, certain forms of light sensitivity have been observed in cells that lack classical photoreceptors, including light-induced calcium waves and the activation of other secondary messenger systems (74, 75). These observations suggest that cells may exhibit sensitivity to weak electromagnetic signals, although the underlying mechanisms are not yet fully understood.

### 5.2 Intercellular photon communication

Numerous experiments support the idea that communication between cells can occur not only through chemical signals but also via weak light emissions. One of the earliest observations, Gurwitch’s mitogenic radiation in the 1920s, suggested that cell proliferation could be influenced by optical interactions between biological samples. More recently, Kobayashi and colleagues (48) demonstrated non-chemical interactions between different plant cell cultures, interactions that were significantly reduced when optical shielding was applied. In more complex systems, such as the nervous and immune systems, some studies suggest that coordinated cellular activity may be associated with photon emission dynamics. This phenomenon could be relevant for processes such as neuromodulation and cell division, although direct mechanistic evidence remains limited (76–78). Overall, these findings suggest that photon emission may accompany coordinated biological processes, but do not yet establish a causal signalling role.

### 5.3 Possible mechanisms

For light-based intercellular communication to occur, two fundamental conditions must be met. First, the emitter cell must be capable of producing photons in a controlled manner, either in response to external stimuli or according to intrinsic rhythmic activity. Second, the receiver cell must be able to detect these photons and respond functionally. Such responses would hypothetically involve activation of intracellular pathways following photon detection, although experimentally validated mechanisms remain largely unidentified. It has been hypothesized that photons could act in paracrine or autocrine-like contexts; however, the extremely low intensity of emission raises significant questions regarding physiological relevance. These considerations highlight the need for cautious interpretation and further experimental validation.

### 5.4 The question of photon patterns and information content

The photons emitted by cells can carry information not only based on their quantity, but also on temporal fluctuations (pulsation, oscillation), spectral characteristics (wavelength dispersion, bands), polarization, and other quantum characteristics. Experimental results indicate that certain photon emission patterns may correlate with specific cell types, developmental stages, or stress conditions (79). This form of pattern-based variation may be analogous, at a conceptual level, to other biological signalling modalities, although its functional role remains to be clarified. These observations support the hypothesis that photon emission may reflect underlying biological organization, even if its direct role in communication remains unproven.

## 6 Present scientific evidence for photon emission as a means of DNA communication

Deoxyribonucleic acid (DNA) is a complex molecule belonging to the group of nucleic acids that stores genetic information. The structure of DNA allows for the stable storage of information, its accurate duplication and its transmission to offspring as hereditary material.

Today’s experimental biology, the understanding and application of the molecular functioning of living systems, is undergoing a huge change. The most important driving force for this is the introduction of the genomic approach. Genomics means genome-scale biology, meaning that studies can extend to the DNA-level or expression (mRNA and/or protein) analysis of all genes of a given organism.

Thanks to this research, our knowledge about the structure and function of DNA has increased tremendously, but our knowledge is mostly based on the results of biochemical research. Despite the fact that ECG, EEG and EMG are widely used not only in research but also in clinical routine, and even the fantastic development of imaging diagnostics has made the use of the results of physics and biophysics as an everyday possibility, we have less faith in this knowledge when it comes to interpreting the basic functioning and functions of cells.

### 6.1 The interaction between photon emission and DNA

Traditionally, macromolecules such as DNA, RNA, and proteins were not considered significant sources of spontaneous light emission under physiological conditions. Pietruszka and Marzec (80) demonstrated that purified DNA samples emit measurable ultra-weak photons under specific experimental conditions. They found that photon emission intensity changed depending on DNA conformational dynamics and oxidative modifications. They observed correlations between DNA relaxation processes and photon emission kinetics and suggested that electronic excitations within DNA molecules may contribute to photon generation. The publication suggested that DNA may not only function as genetic material but could also participate in intracellular electromagnetic phenomena under certain conditions.

However, recent research challenges this concept by demonstrating that under certain conditions and at physiological temperatures, nucleic acids exhibit spontaneous light emission. Through non-invasive observation of barley genomic DNA and advanced statistical physical analyses, temperature-induced dynamic entropy fluctuations and fractal dimensional oscillations were identified at a key organizational threshold. These findings indicate that nucleic acids can contribute to ultra-weak photon emission under specific experimental conditions. The study found evidence of non-equilibrium phase transitions, a noticeable photovoltaic current jump at zero bias, and a proportional increase in photoinduced current with increasing DNA quantity. The results suggest that DNA may represent one of several potential contributors to ultra-weak photon emission in biological systems (80).

DNA forms complex three-dimensional structures through complementary base pairs, whose outstanding feature is high-density information storage—based on its physical dimensions. The theoretical data density of DNA is 6 bits per 1 nm of polymer, or ∼4.5×107 GB/g (81, 82). Therefore, research into the properties of DNA molecules is crucial for biological questions and their application in various industries.

Using Gurwitsch’s findings, Vlail Kaznacheyev conducted thousands of experiments, the results of which were published in book form in 1981 (83). The experiments showed that cellular information can be transmitted electromagnetically and induced in target cells that absorb photon radiation. In the basic experiment, two sealed quartz containers containing the same cell cultures were separated by a thin optical quartz window. One sample was divided into equal parts, and both halves were placed in the two halves of the device, with only a thin optical window visible between them. Thus, the two containers were completely isolated from the environment, except for the optical connection. The cells in one sample were exposed to ionizing radiation, which destroyed them. The cells in the unirradiated culture also died within 12 hours. It was hypothesized that electromagnetic signals, potentially including photon emission, could contribute to the observed effects in target cell cultures.

In another experiment, they used mouse fibroblasts and adult human microvascular endothelial cells, Caco-2 cell lines, and single-celled green algae (Chlamydomonas reinhardtii), all of which exhibited similar optical biocommunication. However, the interpretation of these results remains debated, as alternative explanations such as chemical or environmental influences cannot be fully excluded.

Fritz-Albert Popp discovered a broader spectrum of ultra-weak photon emissions (∼10−3 eV) emitted by living cells, ranging from 200 to 800 nm. He coined the term “biophoton” for these (84). According to Popp et al. (11), a biophoton is a non-thermal photon in the visible and ultraviolet spectrum emitted by a biological system. Popp proposed that biophotons may exhibit coherence-like properties and could play a role in biological regulation, although this remains a theoretical interpretation (85). It has been hypothesized that biophoton emission could be related to processes of electromagnetic interaction within biological systems. He suggested that DNA could play a role in the generation and potential storage of photon energy within cells, although experimental confirmation is still limited (11). He further hypothesized the possibility of large-scale coordination mediated by photon emission, a concept that remains controversial (85).

DNA, as the primary carrier of genetic material, is directly linked to the phenomenon of biophoton emission. The structure of DNA is particularly sensitive to photons and can even be damaged by them, but according to certain theories, they can also serve to transmit information within cells. DNA can act both as a target and, under specific conditions, a potential source of ultra-weak photon emission. Experiments have confirmed that isolated DNA molecules are also capable of emitting photons, especially under stress or when exposed to external electromagnetic fields. Some theoretical models propose that DNA may exhibit resonance-like electromagnetic properties under certain conditions.

Quantum biology assumes that biophoton emission is not merely a by-product of metabolic processes, but part of cell-level communication, and even “light-based” information exchange between cells. Such hypotheses propose coherence-like properties; however, direct empirical validation remains limited.

Biophotons may represent complex communication between cells based on the speed of light. The physics of light seems to fit biological observations. Light is a highly efficient physical carrier of energy and information in physical systems, although its biological utilization at ultra-weak levels remains under investigation. The potential coherence of biophotons may influence signal properties, although its functional role is not yet established. Frequency encoding allows light to encode information from DNA in biophotons. An optical resonator is necessary to store light in a very small, enclosed space. The ability to trap photons and slow down the propagation of light plays an important role in quantum optics (86). The twisting of the light beam can form a propagating spiral pattern that may be capable of reading and encoding parts of DNA and transmitting vast amounts of data (87).

More than fifty years ago, Herbert Fröhlich introduced the concept of long-range coherence in biological systems (88–90). Synchronous, large-scale collective Fröhlich oscillations represent photon emission between cells, which is neither a chemical nor a thermal interaction. The potential coherence of biophotons may influence signal properties, although its functional role is not yet established.

In addition, the spectroscopic properties of the Fröhlich condensate are particularly evident in the narrow linewidth. This ensures long-lived coherence and collective motion of the condensate (91).

Although it has been reported that stochastic fluorescence changes can occur in nucleic acids under the influence of visible light (92), there is no mention in the scientific literature of natural light emission from nucleic acids. This may be due to the fact that the emission of energy quanta can only be detected under certain conditions and only glows for a very short time when the appropriate physical and chemical conditions are present in the body. Therefore, it is likely that previous observations were unsuccessful because the DNA molecule spent most of its time inactive.

B Zhao et al. (93) have identified two dissipative processes in DNA droplets, photoalignment and photofibrillation, as key to cooperatively harnessing stochastic molecular motions. Photofluids can control and amplify nanoscale mechanical motions by orders of magnitude, generating macroscopic, cell-like behaviours including elongation, division, and rotation. These findings demonstrate that light-responsive DNA-based systems can exhibit complex, dynamic behaviour, although their relevance to physiological ultra-weak photon emission remains to be clarified.

The question arises: why does DNA emit light? One possible explanation is that light emission reflects energy redistribution processes at the molecular level. This enables the simultaneous transfer of information in the immediate vicinity of DNA molecules, which may be associated with local molecular interactions, although direct regulatory roles remain hypothetical. In addition, it can trigger a signal transmission cascade that initiates growth in the DNA world under favourable environmental conditions.

## 7 The potential significance of UPE in assisted reproduction

Over the past half century, *in vitro* fertilization (IVF) has undergone significant development and has now become part of everyday medical practice. During the procedure embryo development can be monitored excellently using TimeLapse technology, but this system uses visible light, which can damage cells. All living cells, including embryos, spontaneously emit photons (UPE) generated by metabolic reactions and influenced by physiological conditions. It has been proposed that this phenomenon may provide a basis for monitoring embryo development in a non-invasive and stimulation-free environment. Ultra-weak photon emission has been measured in preimplantation embryos using highly sensitive detection systems under controlled dark conditions. Observations indicate that UPE intensity is lower in degenerated embryos compared to well-developed counterparts. The UPE values of freshly conceived embryos were significantly higher than those of previously frozen and then thawed embryos, but visibly well developing embryos. Differences have also been observed between freshly conceived and cryopreserved embryos, suggesting sensitivity of photon emission to biological condition.

These findings collectively support the development of photon emission–based embryo assessment systems, such as the Photon Emission Embryo Control System (PEECS) (94–97). Importantly, these systems aim to provide non-invasive, label-free evaluation of embryo viability without exposure to external illumination. These observations are consistent with previous reports demonstrating a relationship between metabolic activity and UPE intensity in living systems. Furthermore, differences between viable and degenerated embryos suggest that UPE measurements may provide complementary information to conventional morphological assessment. At present, the biological interpretation of these emission patterns remains preliminary, and further studies combining quantitative photon detection with biochemical and developmental endpoints are required to determine their relationship to embryo viability.

## 8 Discussion

### 8.1 Endocrine implications of ultra‑weak photon emission in reproductive biology

Ultra-weak photon emission (UPE), traditionally interpreted as a secondary consequence of oxidative metabolic processes, acquires additional relevance when examined within an endocrine and reproductive biology framework. At the cellular level, UPE primarily originates from electronically excited molecular species generated during reactive oxygen species (ROS)-mediated reactions, including lipid peroxidation and protein or DNA oxidation. These oxidative pathways are not autonomous processes; rather, they are tightly regulated by endocrine signalling, particularly in hormonally responsive tissues.

In reproductive systems, endocrine regulation exerts a central influence on mitochondrial dynamics, redox homeostasis, and metabolic flux. Gonadotropins, steroid hormones, and local paracrine regulators modulate mitochondrial activity and ROS production through both genomic and non-genomic pathways. Consequently, the biochemical processes underlying UPE are directly embedded within hormonally controlled cellular states. In this respect, UPE can be conceptualized not as an isolated physical phenomenon, but as a functional optical correlate of endocrine-regulated metabolism.

Physiological ROS signalling plays a critical role in key reproductive events, including folliculogenesis, ovulation, fertilization, and early embryonic development. These processes depend on finely tuned redox balance, where both insufficient and excessive ROS levels can compromise cellular integrity and developmental competence. In assisted reproduction, altered redox states are strongly associated with impaired oocyte quality, disrupted fertilization, and suboptimal embryo viability. Given that UPE intensity and temporal dynamics are closely linked to oxidative metabolism, photon emission may provide a sensitive, real-time indicator of these hormonally modulated physiological processes.

Importantly, endocrine regulators themselves contribute to shaping the redox environment. For example, melatonin, a key hormone with well-established roles in circadian and reproductive regulation, exerts potent antioxidant effects and modulates mitochondrial function through receptor-mediated and receptor-independent mechanisms. Through such pathways, hormonal signals influence the generation and decay of excited molecular species that ultimately give rise to photon emission. This establishes a mechanistic continuum from endocrine signalling through redox modulation to measurable UPE.

Within the context of early embryogenesis, where transcriptional activation, mitochondrial reprogramming, and rapid cell division occur under tightly controlled conditions, the integration of metabolic and endocrine signals becomes particularly pronounced. Embryos represent highly dynamic, non-equilibrium systems in which fluctuations in redox state, gene expression, and cellular organization are temporally coordinated. The resulting patterns of UPE may therefore reflect not only instantaneous metabolic activity but also the overall coherence of these regulatory processes. From this perspective, entropy-based and time-resolved analyses of photon emission could capture aspects of developmental competence that emerge from the integration of endocrine and metabolic regulation.

While current evidence does not support the view that UPE constitutes a primary mode of long-range intercellular signalling comparable to classical endocrine pathways, its potential role as a rapid, non-invasive indicator of hormonally regulated cellular states is increasingly compelling. UPE may thus be positioned within a hierarchical framework: (i) as a by-product of oxidative reactions, (ii) as an integrative readout of redox-dependent metabolic activity, and (iii) as a candidate contributor to short-range or intra-system coordination processes within complex biological systems.

Taken together, these considerations suggest that UPE represents the optical reflection of endocrine–metabolic coupling in reproductive biology. By linking hormone-driven regulation of mitochondrial function and oxidative balance with measurable photon emission dynamics, UPE offers a novel perspective on the functional integration of biochemical and biophysical processes. This framework may also provide a rational basis for the development of non-invasive diagnostic approaches, such as photon emission–based embryo assessment systems, while remaining grounded in established principles of endocrine physiology rather than speculative quantum-biological interpretations. In this framework, UPE represents the optical manifestation of hormonally regulated oxidative dynamics rather than an autonomous signalling system.

### 8.2 Future research directions

The ultra-weak photon emission of living cells is an exciting and multidimensional phenomenon that fundamentally affects biological organization, cellular information processing, and even the quantum physical interpretation of life. Although many important observations have been made in recent decades, the functional significance, mechanism, and information content of the phenomenon remain unclear. Among the theoretical frameworks proposed so far, models describing coherent-like biophoton fields and cells as coupled oscillator systems have attracted particular attention, in which the detection of temporal synchronization of cell cultures based on photon emission and the identification of fractal or nonlinear dynamics in emission patterns (e.g., recurrence analysis, detection of chaotic dynamics) appears promising, particularly in relation to temporal synchronization and the identification of nonlinear or fractal-like dynamics in emission patterns (e.g., recurrence analysis and detection of complex temporal structures).

Numerical modelling of coupled oscillator systems, including quantum-inspired extensions of classical models (e.g., modified Kuramoto-type frameworks), represents a promising direction for theoretical research. R. Riera Aroche et al. (98) described how DNA behaves like a quantum computer, defining the quantum states that form the qubit. Josephson-Junction qubits are one of the most promising platforms for quantum computation. While such interpretations remain theoretical, they highlight potential parallels between information processing in biological and physical systems.

**Figure 3:**
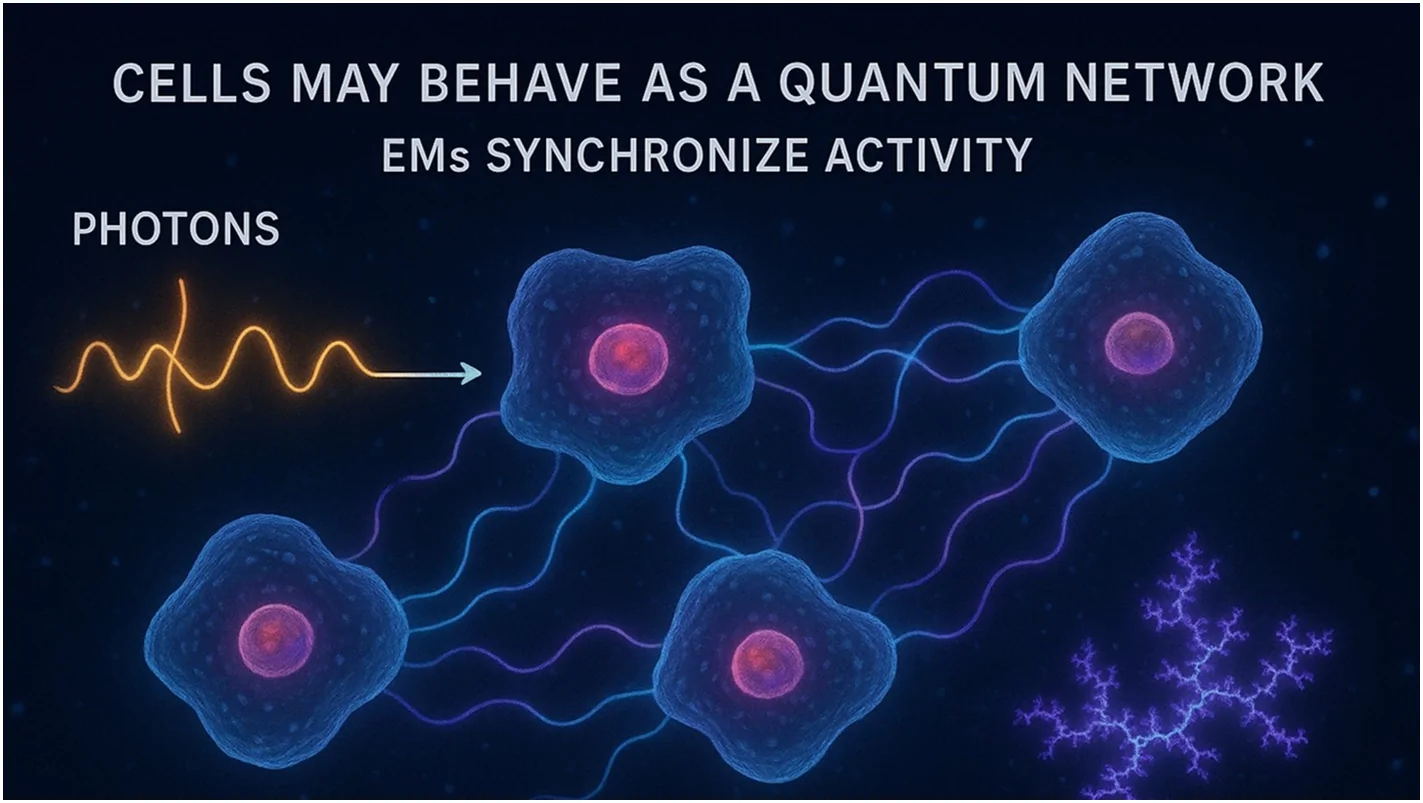
Schematic representation of a quantum communication network between cells. The elementary unit of data carrier is the mediating particle of electromagnetic interaction, the photon. The possible structure of the network shows a self-similar fractal structure, which can be measured based on our studies so far.

The question of photonic communication between cells remains open. Future studies should aim to determine whether photon emission and absorption processes contribute to biologically meaningful interactions between cells. A possible step forward may involve the investigation of photon emission and absorption dynamics in the context of self-similar or fractal-like organizational structures (**Figure 3**). Such approaches may provide insight into whether structured emission patterns reflect underlying biological organization. The underlying concept is that: the structure of the energy emitted by cells may be characteristic of the cellular state and could exhibit self-similar or fractal-like properties. This raises the possibility that emission patterns could be used to differentiate between cellular states or types. If a fraction of emitted energy carries functionally relevant information, then differences in emission structure may be detectable through quantitative analysis. Future research should therefore focus on integrating high-sensitivity detection technologies with advanced analytical methods, including signal processing, information theory, and systems biology approaches. In particular, entropy-based metrics and temporal pattern analysis may provide valuable tools for decoding structured photon emission signals. Such approaches may ultimately enable the development of non-invasive diagnostic methods and contribute to a better understanding of cellular organization and regulation.

### 8.3 Conclusions

Biophotons are generated at the atomic and/or molecular level, and we believe that they perform a well-defined task. Analysis of photon emission from physiologically important molecules, particularly DNA, requires careful interpretation of complex datasets derived from molecular and spectral measurements. These observations may be interpreted as components of a broader biological framework in which photon emission reflects interconnected biochemical and biophysical processes. If we consider that more than seventy years passed between the discovery of the first nucleic acid in white blood cells and the recognition of the structure of DNA in its current sense, then we must be patient in exploring the real background of photon emission and correctly interpreting the data obtained. This historical perspective highlights the importance of cautious interpretation when evaluating emerging multidisciplinary phenomena.

The ultra-weak photon emission of cells, especially the emission mechanisms linked to DNA and their role in communication, remains a challenging but promising area of research. Based on current literature, there is no conclusive empirical evidence that DNA functions as a dedicated biophoton communication system. However, accumulating experimental observations indicate that nucleic acids can participate in ultra-weak photon emission processes under specific conditions. These hypotheses remain noteworthy as they open new perspectives on biological information processing and cellular organization mechanisms (99–101).

These potential contributions underline the importance of continued interdisciplinary research. -– Highly sensitive quantum optical methods and rigorous experimental protocols are necessary for progress in this field of research. In addition, openness to theoretical models—combined with critical evaluation and experimental validation—is essential to advance the field while maintaining scientific rigor.

We would like to conclude our manuscript with a thought from 1941 by Hungarian Nobel Prize winner Albert Szent-Görgyi (102), but rewritten for today: towards the new quantum biology?

## References

[1] Haddock SHD Moline MA Case JF . Bioluminescence in the sea. Ann Rev Mar Sci. (2010) 2:443–93. doi: 10.1146/annurev-marine-120308-081028 21141672

[2] Widder EA . Bioluminescence in the ocean: Origins of biological, chemical, and ecological diversity. Science. (2010). doi: 10.1126/science.1174269 20448176

[3] Al-Handawi MB Polavaram S Kurlevskaya A Commins P Schramm S Carrasco-López C . Spectrochemistry of firefly bioluminescence. Chem Rev. (2022) 122:13207–34. doi: 10.1021/acs.chemrev.1c01047 35926147

[4] Niggli HJ . Ultraweak photons emitted by cells: biophotons. J Photochem Photobiol B. (1992) 14:144–6. doi: 10.1016/1011-1344(92)85090-h 1432381

[5] Devaraj M Martosubroto P . Small pelagic resources and their fisheries in the Asia-Pacific region. In: Proceedings of the APFIC. Bangkok: Asia-Pacific Fishery Commission Food and Agriculture Organization of the United Nations Regional Office for Asia and the Pacific (1997).

[6] Vogel R Süssmuth R . Low level chemiluminescence from liquid culture media. J Appl Microbiol. (1999) 86:999–1007. doi: 10.1046/j.1365-2672.1999.00784.x 10389247

[7] Niggli HJ . Temperature dependence of ultraweak photon emission in fibroblastic differentiation after irradiation with artificial sunligIndian j exp biol. Indian J Exp Biol. (2003) 41:419–23. 15244262

[8] Tafur J Van Wijk EPA Van Wijk R Mills PJ . Biophoton detection and low-intensity light therapy: a potential clinical partnership. Photomed Laser Surg. (2010) 28:23–30. doi: 10.1089/pho.2008.2373 19754267 PMC2957070

[9] Prasad A Pospíšil P . Towards the two-dimensional imaging of spontaneous ultra-weak photon emission from microbial, plant and animal cells. Sci Rep. (2013) 3:1211. doi: 10.1038/srep01211 23386970 PMC3564034

[10] Yan Z Zhang X . Preliminary study of the human body surface photon emission. Prog Biochem Biophysics. (1979) 2:48–52.

[11] Popp FA Nagi W Li KH Scholz W Weingartner O Wolf R . Biophoton emission. New evidence for coherence and DNA as source. Cell Biophys. (1984) 6:33–51. doi: 10.1007/BF02788579 6204761

[12] Cadenas E Siesi H Campa A Cilento G . Electronically excited states in microsomal membranes: Use of chlorophyll-a as an indicator of triplet carbonyls. Photochem Photobiol. 40:661–6. doi: 10.1111/j.1751-1097.1984.tb05356.x 40046247

[13] Quickenden TI Comarmond MJ Tilbury RN . Ultra weak bioluminescence spectra of stationary phase Saccharomyces cerevisiae and Schizosaccharomyces pombe. Photochem Photobiol. (1985). doi: 10.1111/j.1751-1097.1985.tb03534.x 3892556

[14] Inaba H . Super-high sensitivity systems for detection and spectral analysis of ultraweak photon emission from biological cell cells and tissues. Experientia. (1988) 44:550–9. doi: 10.1007/BF01953302 3294030

[15] Gurwitsch AG . Die natur des spezifischen erregers der zellteilung. Dev Genes Evol. (1923) 100:11. doi: 10.1007/bf02111053 30311153

[16] Laager F . Light based cellular interactions: hypotheses and perspectives. Front Phys. (2015) 3. doi: 10.3389/fphy.2015.00055

[17] Quickenden TI Tilbury RN . Luminescence spectra of exponential and stationary phase cultures of respiratory deficient Saccharomyces cerevisiae. J Photochem Photobiol B. (1991) 8:169–74. doi: 10.1016/1011-1344(91)80055-m 1904918

[18] Gallep CM dos Santos SR . Photon-counts during germination of wheat (Triticum aestivum) in wastewater sediment solutions correlated with seedling growth. Seed Sci Technol. (2007) 35:607–14. doi: 10.15258/sst.2007.35.3.08

[19] Cohen S Popp FA . Biophoton emission of human body. Indian J Exp Biol. (2003) 41:440–5. doi: 10.1016/s1011-1344(97)00050-x 15244265

[20] Schwabl H Klima H . Spontaneous ultraweak photon emission from biological systems and the endogenous light field. Forsch Komplementarmed Klass Naturheilkd. (2005) 12:84–9. doi: 10.1159/000083960 15947466

[21] Pospíšil P Prasad A Rác M . Mechanism of the formation of electronically excited species by oxidative metabolic processes: role of reactive oxygen species. Biomolecules. (2019) 9:E258. doi: 10.3390/biom9070258 31284470 PMC6681336

[22] Prasad A Mihačová E Manoharan RR Pospíšil P . Application of ultra-weak photon emission imaging in plant stress assessment. J Plant Res. (2025) 138:389–400. doi: 10.1007/s10265-024-01600-w 39757329 PMC11910446

[23] Du J Deng T Cao B Wang Z Yang M Han J . The application and trend of ultra-weak photon emission in biology and medicine. Front Chem. (2023) 11:1140128. doi: 10.3389/fchem.2023.1140128 36874066 PMC9981976

[24] Nevoit G Poderiene K Potyazhenko M Mintser O Jarusevicius G Vainoras A . The concept of biophotonic signaling in the human body and brain: rationale, problems and directions. Front Syst Neurosci. (2025) 19:1597329. doi: 10.3389/fnsys.2025.1597329 40626233 PMC12230014

[25] Cifra M Pospíšil P . Ultra-weak photon emission from biological samples: Definition, mechanisms, properties, detection and applications. J Photochem Photobiol B Biol. (2014) 139:2–10. doi: 10.1016/j.jphotobiol.2014.02.009 24726298

[26] Pospíšil P Prasad A Rac M . Role of reactive oxygen species in ultra-weak photon emission in biological systems. J Photochem Photobiol B. (2014) 139:11–23. doi: 10.1016/j.jphotobiol.2014.02.008 24674863

[27] Pospíšil P Yamamoto Y . Damage to photosystem II by lipid peroxidation products. Biochim Biophys Acta Gen Subj. (2017) 1861:457–66. doi: 10.1016/j.bbagen.2016.10.005 27741410

[28] Shanei A Alinasab Z Kiani A Nematollahi MA . Detection of ultraweak photon emission (UPE) from cells as a tool for pathological studies. J BioMed Phys Eng. (2017) 7:389–96. PMC5809932 29445715

[29] Ayala A Muñoz MF Argüelles S . Lipid peroxidation: production, metabolism, and signaling mechanisms of malondialdehyde and 4-hydroxy-2-nonenal. Oxid Med Cell Longev. (2014) 2014:360438. doi: 10.1155/2014/360438 24999379 PMC4066722

[30] Bucher JR . Oxidative stress and radical-induced signalling. In: Baan RA Stewart BW Straif K , editors. Iarc Scientific Publications, No. 165. International Agency for Research on Cancer, Lyon (FR (2019). 33979089

[31] Kaznacheev AVP Mikhailova LP Kartashov NB . Distant intercellular electromagnetic interaction between two tissue cultures. Bull Exp Biol Med. (1980) 89:345–8. doi: 10.1007/BF00834249 6248145

[32] Cifra M Fields JZ Farhadi A . Electromagnetic cellular interactions. Prog Biophys Mol Biol. (2011) 105:223–46. doi: 10.1016/j.pbiomolbio.2010.07.003 20674588

[33] Cifra M Brouderb C Nerudov M . Biophotons, coherence and photocount statistics: a critical review. arXiv:1502.07316v1 [physics.bio-ph]. (2015). doi: 10.1016/j.jlumin.2015.03.020 38826717

[34] Mould RR Mackenzie AM Kalampouka I Nunn AVW Thomas EL Bell JD . Ultra weak photon emission a brief review. Front Physiol. (2024) 15:1348915. doi: 10.3389/fphys.2024.1348915 38420619 PMC10899412

[35] Nowak M Sasak K Wlodarczyk A Grabska-Kobylecka I Sarniak A Nowak D . Ultra-weak photon emission from crown ethers exposed to Fenton's reagent Fe2+-H2O2. Molecules. (2025) 30:3282. doi: 10.3390/molecules30153282 40807454 PMC12348572

[36] Bat’yanov AP . Distant-optical interaction of mitochondria through quartz. Biull Eksp Biol Med. (1984) 97:675–7. doi: 10.1007/BF00804160 6331545

[37] Scholkmann F Fels D Cifra M . Non-chemical and non-contact cell-to-cell communication: a short review. Am J Transl Res. (2013) 5:586–93. PMC3786266 24093056

[38] Thar R Kühl M . Propagation of electromagnetic radiation in mitochondria? J Theor Biol. (2004) 230:261–70. doi: 10.1016/j.jtbi.2004.05.021 15302557

[39] Craddock TJA Friesen D Mane J Hameroff S Tuszynski JA . The feasibility of coherent energy transfer in microtubules. J R Soc Interface. (2014) 11:20140677. doi: 10.1098/rsif.2014.0677 25232047 PMC4191094

[40] Prasad A Pospíšil P . Linoleic acid-induced ultra-weak photon emission from Chlamydomonas reinhardtii as a tool for monitoring of lipid peroxidation in the cell membranes. PloS One. (2011) 6:e22345. doi: 10.1371/journal.pone.0022345 21799835 PMC3143142

[41] Nunn AVW Guy GW Bell JD . Bioelectric fields at the beginnings of life. Bioelectricity. (2022) 4:237–47. doi: 10.1089/bioe.2022.0012 36636557 PMC9810354

[42] Dhalla NS Temsah RM Netticadan T . Role of oxidative stress in cardiovascular diseases. J Hypertens. (2000) 18:655–73. doi: 10.1097/00004872-200018060-00002 10872549

[43] Rodriguez R Redman R . Balancing the generation and elimination of reactive oxygen species. PNAS. (2005). doi: 10.1073/pnas.0500367102 15728396 PMC552941

[44] Newsholme P Fernandes Cruzat V Keane KN Carlessi R Homem de Bittencourt PI . Molecular mechanisms of ROS production and oxidative stress in diabetes. Biochem J. (2016) 473:4527–50. doi: 10.1042/BCJ20160503C 27941030

[45] Prasad S Gupta SC Tyagi AK . Reactive oxygen species (ROS) and cancer: Role of antioxidative nutraceuticals. Cancer Lett. (2017) 387:95–105. doi: 10.1016/j.canlet.2016.03.042 27037062

[46] Phull AR Nasir B Haq IU Kim SJ . Oxidative stress, consequences and ROS mediated cellular signaling in rheumatoid arthritis. Chem Biol Interact. (2018) 281:121–36. doi: 10.1016/j.cbi.2017.12.024 29258867

[47] Sumien N Cunningham JT Davis DL Engelland R Fadeyibi O Farmer GE . Neurodegenerative disease: Roles for sex, hormones, and oxidative stress. Endocrinology. (2021) 162:bqab185. doi: 10.1210/endocr/bqab185 34467976 PMC8462383

[48] Kobayashi M Takeda M Sato T Yamazaki Y Kaneko K Ito KI . *In vivo* imaging of spontaneous ultraweak photon emission from a rat's brain correlated with cerebral energy metabolism and oxidative stress. Neurosci Res. (1999) 34:103–13. doi: 10.1016/S0168-0102(99)00040-1 10498336

[49] Paolis LD Francini R Davoli I De Matteis F Scordo A Clozza A . Biophotons: A hard problem. Appl Sci. (2024) 14:5496. doi: 10.3390/app14135496 30654563

[50] Prasad A Gouripeddi P Devireddy HRN Ovsii A Rachakonda DP Wijk RV . Spectral distribution of ultra-weak photon emission as a response to wounding in plants: An *in vivo* study. Biology. (2020) 9:139. doi: 10.3390/biology9060139 32604795 PMC7345010

[51] Naumova EV Vladimirov YA Tuchin VV Namiot VA Volodyaev IV . Methods of studying ultraweak photon emission from biological objects: III. Physical methods. Biophysics. (2022) 67:27–58. doi: 10.1134/S0006350922010109

[52] Nevoit G Bumblytė IA Korpan A Minser O Potyazhenko M Iliev MT . The biophoton emission in biotechnological and chemical research: from meta-epistemology and meaning to experiment. Part 1. Ukr J Phys. (2024) 69:190–206. doi: 10.15407/ujpe69.3.190

[53] Kobayashi M Sasaki K Enomoto M Ehara Y . Highly sensitive determination of transient generation of biophotons during hypersensitive response to cucumber mosaic virus in cowpea. J Exp Bot. (2006) 58:465–72. doi: 10.1093/jxb/erl215 17158510

[54] Photonis . Photomultiplier tube basics (2002). Available online at: https://psec.uchicago.edu/library/photomultipliers/Photonis_PMT_basics.pdf (Accessed April 11, 2023).

[55] Chen YS Chen BT . Measuring of a three-dimensional surface by use of a spatial distance computation. Appl Optics. (2003) 42:1958–72. doi: 10.1364/ao.42.001958 12699342

[56] Zhang L Neves L Lundeen JS Walmsley IA . A characterization of the single-photon sensitivity of an electron multiplying charge-coupled device. J Phys B: Atomic Mol Optical Phys. 42. doi: 10.1088/0953-4075/42/11/114011

[57] Saeidfirozeh H Shafiekhani A Cifra M Masoudi AA . Endogenous chemiluminescence from germinating Arabidopsis thaliana seeds. Sci Rep. (2018) 8:16231. doi: 10.1038/s41598-018-34485-6 30385859 PMC6212569

[58] Khaoua I Graciani G Kim A Amblard F . Detectivity optimization to measure ultraweak light fluxes using an EM-CCD as binary photon counter array. Sci Rep. (2021) 11:3530. doi: 10.1038/s41598-021-82611-8 33574351 PMC7878522

[59] Kurtsiefer C Oberparleiter M Weinfurter H . High-efficiency entangled photon pair collection in type-II parametric fluorescence. Phys Rev A. (2001) 64:23802. doi: 10.1103/physreva.64.023802 39592951

[60] Mould RR Mackenzie AM Kalampouka I Nunn AVW Thomas EL Bell JD . Ultra weak photon emission—a brief review. Front Physiol. (2024) 15:1348915. doi: 10.3389/fphys.2024.1348915 38420619 PMC10899412

[61] Yang M Zhang Z Fu J Liu J Pang J Fan H . Ultra-weak photon emission as a potential tool for evaluating the therapeutic effect of traditional Chinese medicine in patients with type 2 diabetes. Heliyon. (2023) 9:e18055. doi: 10.1016/j.heliyon.2023.e18055 37519692 PMC10372244

[62] Popp F-A . Photon storage in biological systems. In: Popp F-A Becker G Koenig HL Peschka W , editors. Electromagnetic Bio-Information. Urban & Schwarzenberg, Munich (1979). p. 123–48.

[63] Popp F-A . Properties of biophotons and their theoretical implications. Indian J Exp Biol. (2003) 41:391–402. 15244259

[64] Benfatto M Pace E Davoli I Francini R De Matteis F Scordo A . Biophotons: New experimental data and analysis. Entropy. (2023) 25:1431. doi: 10.3390/e25101431 37895552 PMC10606557

[65] Engel GS Calhoun TR Read EL Ahn T-K Mancal T Cheng Y-C . Evidence for wavelike energy transfer through quantum coherence in photosynthetic systems. Nature. (2007) 446:782–6. doi: 10.1038/nature05678 17429397

[66] Ritz T . Quantum effects in biology: Bird navigation. Proc Chem. (2011) 3:262–75. doi: 10.1016/j.proche.2011.08.034 38826717

[67] Holland RA . True navigation in birds: from quantum physics to global migration. J Zool. (2014). doi: 10.1111/jzo.12107 40046247

[68] Sagar SM Thomas RJ Loverock LT Spittle MF . Olfactory sensations produced by high-energy photon irradiation of the olfactory receptor mucosa in humans. Int J Radiat Oncol Biol Phys. (1991) 20:771–6. doi: 10.1016/0360-3016(91)90021-u 1822956

[69] Bandari A . Human smell perception is governed by quantum spin-residual information. Scilight News. (2019) 2019. doi: 10.1063/1.5121155

[70] Willeford K . The luminescence hypothesis of olfaction. Sensors (Basel). (2023) 23:1333. doi: 10.3390/s23031333 36772376 PMC9919928

[71] Karu T . Primary and secondary mechanisms of action of visible to near-IR radiation on cells. J Photochem Photobiol B. (1999) 49:1–17. doi: 10.1016/S1011-1344(98)00219-X 10365442

[72] Jibu M Yasue K Hagan S . Evanescent (tunneling) photon and cellular ‘vision’. BioSystems. (1997) 42:65–73. doi: 10.1016/s0303-2647(97)01686-9 9146835

[73] Hameroff S Penrose R . Orchestrated reduction of quantum coherence in brain microtubules: a model for consciousness. Math Comput Simul. (1996) 40:453–80. doi: 10.1016/0378-4754(96)80476-9

[74] Kučera O Cifra M . Cell-to-cell signaling through light: just a ghost of chance? Cell Commun Signal. (2013) 11:87. doi: 10.1186/1478-811X-11-87 24219796 PMC3832222

[75] Mould RR Kalampouka I Thomas EL Guy GW Nunn AVW Bell JD . Non-chemical signalling between mitochondria. Front Physiol. (2023) 14:1268075. doi: 10.3389/fphys.2023.1268075 37811497 PMC10560087

[76] Tang R Dai J . Biophoton signal transmission and processing in the brain. J Photochem Photobiol B. (2014) 139:71–5. doi: 10.1016/j.jphotobiol.2013.12.008 24461927

[77] Kumar S Boone K Tuszyński J Barclay P Simon C . Possible existence of optical communication channels in the brain. Sci Rep. (2016) 6:36508. doi: 10.1038/srep36508 27819310 PMC5098150

[78] Esmaeilpour T Fereydouni E Dehghani F Bókkon I Panjehshahin M-R Császár-Nagy N . An experimental investigation of ultraweak photon emission from adult murine neural stem cells. Sci Rep. (2020) 10:463. doi: 10.1038/s41598-019-57352-4 31949217 PMC6965084

[79] Van Wijk EP Van Wijk R Bosman S . Using ultra-weak photon emission to determine the effect of oligomeric proanthocyanidins on oxidative stress of human skin. J Photochem Photobiol B Biol. (2010) 98:199–206. doi: 10.1016/j.jphotobiol.2010.01.003 20138538

[80] Pietruszka M Marzec M . Ultra-weak photon emission from DNA. Sci Rep. (2024) 14:28915. doi: 10.1038/s41598-024-80469-0 39572702 PMC11582580

[81] Knappe GA Wamhoff EC Bathe M . Functionalizing DNA origami to investigate and interact with biological systems. Nat Rev Mater. (2022) 8:123–38. doi: 10.1038/s41578-022-00517-x 37206669 PMC10191391

[82] Organick L Chen Y-J Dumas Ang S Lopez R Liu X Strauss K . Probing the physical limits of reliable DNA data retrieval. Nat Commun. (2020) 11:616. doi: 10.1038/s41467-020-14319-8 32001691 PMC6992699

[83] Kaznacheev VP Mihaylova MP . Ultra-weak radiations in intercellular interactions. Science Novosibirsk. (1981).

[84] Bischof M . Introduction to integrative biophysics. In: Popp F-A Beloussov L , editors. Integrative Biophysics. Springer, Dordrecht (2003). p. 1–115. doi: 10.1007/978-94-017-0373-4_1

[85] Popp FA Li KH Mei WP Galle M Neurohr R . Physical aspects of biophotons. Experientia. (1988) 44:576–85. doi: 10.1007/bf01953305 3294033

[86] Tanabe T Notomi M Kuramochi E Shinya A Taniyama H . Trapping and delaying photons for one nanosecond in an ultrasmall high-Q photonic-crystal nanocavity. Nat Photonics. (2007) 1:49–52. doi: 10.1038/nphoton.2006.51 37880705

[87] Feldmann A Ivanek R Murr R Gaidatzis D Burger L Schübeler D . Transcription factor occupancy can mediate active turnover of DNA methylation at regulatory regions. PloS Genet. (2013) 9:e1003994. doi: 10.1371/journal.pgen.1003994 24367273 PMC3868540

[88] Fröhlich H . Long-range coherence and energy storage in biological systems. Int J Quantum Chem. (1968) 2:641–9. doi: 10.1002/qua.560020505

[89] Vasconcellos ÁR Vannucchi FS Mascarenhas S Luzzi R . Fröhlich condensate: Emergence of synergetic dissipative structures in information processing biological and condensed matter systems. Information. (2012) 3:601–20. doi: 10.3390/info3040601 30654563

[90] Nakamura Y Pashkin YA Tsai JS . Coherent control of macroscopic quantum states in a single-Cooper-pair box. Nature. (1999) 398:786–8. doi: 10.1038/19718

[91] Zhang Z Agarwal GS Scully MO . Quantum fluctuations in the Fröhlich condensate of molecular vibrations driven far from equilibrium. Phys Rev Lett. (2019) 122:158101. doi: 10.1103/physrevlett.122.158101 31050540

[92] Dong B Almassalha LM Stypula-Cyrus Y Urban BE Chandler JE Nguyen T-Q . Superresolution intrinsic fluorescence imaging of chromatin utilizing native, unmodified nucleic acids for contrast. Proc Natl Acad Sci USA. (2016) 113:9716–21. doi: 10.1073/pnas.1602202113 27535934 PMC5024634

[93] Zhao Q-H Qi J-Y Deng N-N . DNA photofluids show life-like motion. Nat Mater. (2025) 24:935–44. doi: 10.1038/s41563-025-02202-0 40204968

[94] Berke J Gulyás I Bognár Z Berke D Enyedi A Kozma-Bognár V . Unique algorithm for the evaluation of embryo photon emission and viability. Sci Rep. (2024) 14:15066. doi: 10.1038/s41598-024-61100-8 38956113 PMC11220017

[95] Bodis J Nagy B Bognar Z Csabai T Berke J Gulyas I . Detection of ultra-weak photon emissions from mouse embryos with implications for assisted reproduction. J Health Care Commun. (2024) 9.

[96] Shannon CE . A mathematical theory of communication. Bell Syst Tech J. (1948) 27:379–423. doi: 10.1145/584091.584093

[97] Shannon CE . Prediction and entropy of printed English. Bell Syst Tech J. (1951) 30:50–64. doi: 10.1002/j.1538-7305.1951.tb01366.x 41531421

[98] Riera Aroche R Ortiz García YM Martínez Arellano MA Riera Leal A . DNA as a perfect quantum computer based on the quantum physics principles. Sci Rep. (2024) 14:11636. doi: 10.1038/s41598-024-62539-5 38773193 PMC11109248

[99] Popp FA Li KH Gu Q . Recent advances in biophoton research and its aplication. In: World Scientific. Singapore City, London, New York: Springer Nature (1992). doi: 10.1142/1559

[100] Choi KH Lee JH Kim MY Kim JH Lim J Lee J . Effect of 710 nm visible light irradiation on neurite outgrowth in primary rat cortical neurons following ischemic insult. Biochem Biophys Res Commun. (2012) 422:274–9. doi: 10.1016/j.bbrc.2012.04.147 22580279

[101] Van Wijk R Van Wijk EPA . An introduction to human biophoton emission. Forsch Komplementärmed Klass Naturheilkd. (2005) 12:77–83. doi: 10.1159/000083763 15947465

[102] Szent-Györgyi A . Towards a new biochemistry? Science. (1941) 93:609–11. doi: 10.1126/science.93.2426.609 17841996

[103] Prasad A Pospíšil A . Towards the two-dimensional imaging of spontaneous ultra-weak photon emission from microbial, plant and animal cells. Scientific Reports. (2013) 3:1211. 23386970 10.1038/srep01211 PMC3564034

